# Is There a “Gifted Personality”? Initial Evidence for Differences between MENSA and General Population Members in the HEXACO Personality Inventory

**DOI:** 10.3390/jintelligence10040092

**Published:** 2022-10-26

**Authors:** Jonathan Fries, Kristof Kovacs, Elisabeth L. Zeilinger, Jakob Pietschnig

**Affiliations:** 1 Department of Developmental and Educational Psychology, Faculty of Psychology, University of Vienna, 1010 Vienna, Austria; 2 Institute of Psychology, ELTE Eotvos Lorand University Budapest, 1053 Budapest, Hungary; 3 Clinical Division of Palliative Medicine, Department of Internal Medicine I, Medical University of Vienna, 1090 Vienna, Austria

**Keywords:** giftedness, personality, cognitive ability, HEXACO, group differences

## Abstract

Contrary to the common notion that personality and intelligence are unrelated constructs, numerous correlational studies have demonstrated substantial associations between the two domains. Moreover, samples of intellectually gifted individuals have been found to differ from the general population in specific aspects of their personalities. However, most studies so far have relied on the Five-Factor Model of Personality (FFM), while none have investigated this phenomenon using the HEXACO personality framework. We recruited 617 adult members of the international high-IQ society MENSA and compared them to 3 reference samples (combined *N* = 112,637) regarding their personalities as measured by the HEXACO-60 personality inventory. We found that gifted persons scored higher in Honesty-Humility and Conscientiousness but lower in Emotionality compared to reference samples. Interestingly, gifted individuals scored only slightly higher in Openness to Experience, and no consistent differences emerged for Agreeableness. We demonstrate that some known personality differences between gifted and non-gifted persons translate from the FFM to the HEXACO model, while others do not. Our results indicate that within the HEXACO factor structure differences in sociability are more pronounced, while intellect-related differences are comparatively weak.

## 1. Introduction

Historically, intelligence and personality have been regarded as independent constructs (e.g., Eysenck 1971). However, over the past decades, evidence has accumulated that suggests otherwise. Many studies have found non-trivial associations between measures of intelligence and various personality constructs (Ackerman and Heggestad 1997; DeYoung 2020; Stanek 2014), thus suggesting a potential relation of these psychological domains.

In the study of the intelligence–personality association, investigations have so far typically used correlational designs, thus modelling linear relationships between intelligence and personality variables (Ashton et al. 2000; Chamorro-Premuzic and Furnham 2008; Kretzschmar et al. 2018). However, there have been justified concerns about the generalizability of these correlational findings to the upper end of the intelligence distribution, because effects at the extreme ends of the intelligence distribution may behave in a different manner than in the center of the distribution (Wirthwein et al. 2019). Individuals within this range are often referred to as intellectually gifted. Most definitions agree that giftedness is characterized by exceptional cognitive ability which is usually measured by standardized intelligence test batteries (Baudson 2016). A common cut-off value for intellectual giftedness is a score that places the individual at least two standard deviations above the general population mean (Warne 2015). There is less agreement among scholars regarding components that identify giftedness apart from intellectual excellence (Carman 2013), the most commonly cited elements being achievement motivation and creativity. However, arguably, achievement motivation is a necessary precondition for performing well on an intelligence test, thus making separate assessments redundant, whilst creativity tests are typically criticized for unsatisfactory validities (Kaufman et al. 2012; Almeida et al. 2008). Consequently, for the purposes of the present study, we adopt a purely intelligence-based definition of giftedness (i.e., adopting a lower threshold of 130 in the IQ metric, which indicates individuals scoring at least two standard deviations above the mean).

To examine the specific personality differences between intellectually gifted and non- gifted individuals, commonly sampled scores of gifted individuals are compared with those of non-gifted controls. In our literature review, we present studies that adopted correlational as well as studies that adopted group comparison designs.

### 1.1. Personality

Constructed using a lexical approach, the Five-Factor Model of Personality (FFM; Digman 1990; McCrae and Costa 1987) is, to date, arguably the most popular framework in personality research (McCrae and Costa 2008). It consists of the orthogonal factors Openness to Experience, Conscientiousness, Extraversion, Agreeableness, and Neuroticism. Most of the research about a possible intelligence–personality link has been carried out using different measures of the FFM (Stanek 2014). 

Similar to the FFM, the HEXACO personality model is also based on lexical methodology, but, in contrast to the FFM, a multi-language adjective pool was used for its development, whereas the FFM was derived from the English language. This inclusion of additional linguistic content led to the emergence of a sixth orthogonal factor in the HEXACO model instead of the traditional five factors (Lee et al. 2005a). It has been argued that this six-factor solution is superior in terms of interpretability (Lee and Ashton 2004) and that it exhibits higher validity in predicting behavioral outcomes (e.g., Lee and Ashton 2005; Lee et al. 2005b) compared to the FFM. Superficially, the main differences between these two taxonomies is the addition of a sixth factor (i.e., Honesty-Humility) in the HEXACO, whilst the remaining five factors are essentially labelled identically to the FFM factors. However, the HEXACO factor structure represents, in fact, a revised factor structure of the FFM model by repartitioning variance of the FFM agreeableness and neuroticism factors into the HEXACO factors of agreeableness, emotionality, and honesty–humility. This means that the five and six factors of the FFM and HEXACO model are—albeit having identical labels—characterized to a certain extent by different lower-order facets (e.g., Anglim and O’Connor 2019).

Thus, the HEXACO model of personality consists of the six factors of Honesty-Humility, Emotionality, Extraversion, Agreeableness, Conscientiousness, and Openness to Experience (henceforth: Openness). Each factor has four hierarchically subordinate facets that reflect more specific aspects of personality (Ashton and Lee 2009). In Table 1, we provide brief descriptions of all HEXACO factors and facets.

### 1.2. Personality and Cognitive Ability

Only the two FFM personality factors, Openness and Neuroticism, have so far been shown to yield systematic and reproducible links with giftedness. Among FFM factors, Openness has been reported to show the largest, most consistent associations with cognitive ability. In a meta-analysis on the association between intelligence and personality, a summary effect size of *r* = .33 was reported for the correlation between general intelligence and FFM Openness (Ackerman and Heggestad 1997). Subsequent meta-analyses came to similar estimates of *r* = .30 (DeYoung 2011) and *r* = .25 (Stanek 2014). These results indicate that higher intelligence is associated with a higher degree of Openness. Studies among intellectually gifted adolescents point in the same direction, yielding higher scores of Openness for intellectually gifted adolescents compared to the general population (*d* = 0.56, Limont et al. 2014; *d* = 0.61, Wirthwein et al. 2019). Recent meta-analytic evidence has found a similar effect size in the same direction for Openness (*d* = 0.47, Ogurlu and Özbey 2021).

In contrast, Neuroticism has been reported to be negatively associated with intelligence, with effect sizes ranging from *r* = −.10 (Ackerman and Heggestad 1997) to *r* = −.15 (Stanek 2014). This indicates that higher intelligence tends to come with lower Neuroticism. When compared to non-gifted students, intellectually gifted students scored lower in Neuroticism (*d* = −0.26; Zeidner and Shani-Zinovich 2011). Group comparisons yielded lower Neuroticism scores in gifted compared to non-gifted adolescents (*d* = −0.72; Limont et al. 2014). However, such differences were observed to be merely trivial in other studies (*d* = −0.11; Wirthwein et al. 2019), although the effect direction remained consistent. A recent meta-analysis found a meaningful, albeit non-significant, mean difference (*d* = −0.34, Ogurlu and Özbey 2021).

Extraversion has not been observed to exhibit meaningful associations with cognitive ability in meta-analyses, yielding *r* = −.04 (Wolf and Ackerman 2005) or *r* = .08 (Ackerman and Heggestad 1997). Examinations of group differences between gifted and non-gifted individuals yielded mixed results that differed in terms of the effect direction (*d* = 0.06 for adolescents, Wirthwein et al. 2019; *d* = −0.07, Limont et al. 2014). However, in an investigation comparing MENSA members to non-gifted controls, significantly lower levels of extraversion were reported (*d* = −0.34, Dijkstra et al. 2012). Meta-analytic evidence indicates a small, positive effect (*d* = 0.18, Ogurlu and Özbey 2021).

Conscientiousness and Agreeableness have been reported to be merely negligibly associated with intelligence. Meta-analyses reported effect sizes of *r* = .02 to *r* = −.04 for the association of Conscientiousness with intelligence (Ackerman and Heggestad 1997; Stanek 2014) and effect sizes of *r* = .01 to *r* = −.03 for the association of Agreeableness with intelligence (Ackerman and Heggestad 1997; Stanek 2014). However, Conscientiousness has been found to be elevated in gifted individuals in some studies (*d* = 1.69, Sadat et al. 2014; *d* = 0.20, Biedroń 2011), while the effect did not emerge in others (*d* = 0.06, Wirthwein et al. 2019). A meta-analysis found small and non-significant, positive effects for Conscientiousness (*d* = 0.22) as well as Agreeableness (*d* = 0.17, Ogurlu and Özbey 2021).

### 1.3. The Current Study

Some authors have argued that the HEXACO six-factor structure is psychometrically superior compared to the FFM (Ashton and Lee 2007; Ashton et al. 2014; Zettler et al. 2020). Since the interpretation of the factors and facets of the HEXACO differ in some important regards from the FFM (Lee and Ashton 2004), our goal in the current study was to investigate the pattern of differences in personality between gifted and non-gifted individuals when using the HEXACO model. This is the first study that examines HEXACO-based personality differences between gifted and non-gifted individuals.

We hypothesized that in comparison to the general population, gifted individuals would exhibit lower scores in the HEXACO factors of Emotionality and Extraversion and higher scores in Openness, but no differences in Agreeableness or Conscientiousness. Moreover, we explored potential differences in Honesty-Humility.

## 2. Materials and Methods

Data for the current study were collected as part of another project. Hypotheses were preregistered prior to data analysis (https://aspredicted.org/mv2i2.pdf, accessed on 30 June 2022). 

### 2.1. Sample

In all, 617 individuals (308 women) participated in the current study. The sample was recruited from the MENSA society, an international association of people that have scored at or above the 98th percentile in a psychometrically valid test of cognitive ability. Using a standardized letter, we invited members from the Austrian, German, Hungarian, Swiss, and United Kingdom chapters to partake in our online survey. Potential subjects were approached via official MENSA mailing lists or Facebook groups by MENSA officers. MENSA has more male than female members (American MENSA 2022). However, the typically greater willingness of women to partake in surveys may have counteracted the gender disparity (Feveile et al. 2007). Sample characteristics are detailed in Table 2 and Table 3.

### 2.2. Materials

Participants were asked to respond to an online survey which took approximately 45 to 60 min to finish. The survey comprised sections covering sociodemographic characteristics as well as questionnaires about physical health, mental health, and different aspects of behavior and personality. Subjects were able to choose between an English, German, and Hungarian version of the survey.

Sociodemographic characteristics included age, sex, country of residence, as well as the level of education following the ISCED-2011 framework (UNESCO Institute for Statistics 2012). Occupation was assessed using the ISCO-08 classification (International Labour Office 2012). Moreover, participants were asked to provide their IQ test scores from their MENSA admission test.

Personality was assessed using the HEXACO-60 personality inventory (Ashton and Lee 2009). The HEXACO model describes personality on six distinct factors: Honesty-Humility, Emotionality, Extraversion, Agreeableness, Conscientiousness, and Openness to Experience, which provide another four facet scores describing more particular (correlated) aspects of personality and behavior. The questionnaire consists of 60 items, with 10 pertaining to each of the 6 factors. In each item, subjects were asked to state their level of agreement on a 5-point Likert-typed scale (1 = strongly disagree to 5 = strongly agree; example item: “I would be quite bored by a visit to an art gallery”; Ashton and Lee 2009, p. 345). In the current data, factors showed modest to adequate reliabilities (Cronbach’s α ranging from .67 to .82).

### 2.3. Reference Data

To enable comparisons between our MENSA scores with reference data of samples originating from the entire cognitive ability distribution, we obtained unpublished facet-level summary statistics for three large datasets from one of the authors of the HEXACO (Ashton 2022). We decided to use three different reference samples to investigate whether potential personality differences between gifted and non-gifted individuals consistently emerge across heterogenous participant groups.

The first reference sample was taken from a cross-cultural personality assessment study which examined measurement invariance in the HEXACO model across 16 different languages (Thielmann et al. 2020). Ashton provided us with detailed summary statistics of the German-speaking portion of the sample that were not reported in the published study. The sample was composed of 9491 persons (7263 women) with a mean age of 32.40 years (*SD* = 9.40). In all, 5.81 percent of the sample were high school students over 18 years of age, 20.15 percent were employees with undisclosed educational backgrounds, 0.87 percent were employees with university degrees, and 22.79 percent were university students. For 50.38 percent of the sample, no educational information was available. Cronbach’s α for the multinational sample ranged from .80 to .85 on factor level. This sample will henceforth be referred to as the “Thielmann sample”. In our analyses, we compared the Thielmann sample with the entirety of the MENSA sample. In addition, we ran additional exploratory tests using only the German-speaking portion of the MENSA sample.

The second reference sample was recruited by Lee and Ashton (2018) via an online self-assessment of personality and was composed of 100,318 persons (48,562 women) with a mean age of 37.10 years (*SD* = 14.10). In all, 19.20 percent of participants reported their highest level of education as having finished high school, 41.60 percent college or university, and 32.80 percent graduate or professional school. The English version of the HEXACO was used. Participants were self-selected. The questionnaire contained additional control items to ensure attentiveness. In addition, the authors filtered out implausible response patterns. Cronbach’s α ranged from .82 to .89 on factor level. This sample will henceforth be referred to as the “online sample”.

The third reference sample was also taken from Lee and Ashton (2018). This sample comprised undergraduate students that had provided self- and observer-report personality data. Only self-report data were used as reference. The undergraduate student sample was composed of 2868 persons (1843 women) with a mean age of 20.90 years (*SD* = 4.70). In all, 5.81 percent of the sample were high school students over 18 years of age, 20.15 percent were employees with undisclosed educational backgrounds, 0.87 percent were employees with university degrees, and 22.79 percent were university students. For 50.38 percent of the sample, no educational information was available. Cronbach’s α ranged from .81 to .84 on factor level. This sample will henceforth be referred to as the “student sample”.

In all three reference samples, subjects responded to the 100-item version of the HEXACO. We obtained the data for the 60-item HEXACO, which is a subset of the longer version (Ashton 2022; reported reliabilities pertain to the 100-item questionnaire).

### 2.4. Statistical Analysis

To compare the intellectually gifted MENSA sample to the reference data, we performed independent-sample *t*-tests for all factors and corresponding facets. The MENSA sample was compared to the Thielmann sample, the online sample, and the student sample. In exploratory analyses, we compared the German-speaking portion of the MENSA sample to the Thielmann sample. Since group sizes and variances differed substantially, we computed Welch’s unequal variances *t*-tests. For each comparison, Cohen’s *d* was calculated using the formula for the standardized mean difference by Lipsey and Wilson (2001).

We focus presently on the interpretation of effect sizes according to well-established thresholds (i.e., small, moderate, and large effects having lower thresholds of absolute Cohen d’s of .2, .5, and .8, respectively; Cohen 1988), instead of nominal values from traditional null hypothesis significance testing. Exact *p*-values can be found in the supplementary material. The benchmarks by Nunnally and Bernstein (1994) were adopted for the interpretation of Cronbach’s α.

All data analyses were carried out using R 4.1.0 (R Core Team 2022). Figures were created using ggplot2 (Wickham 2016).

## 3. Results

**Figure 1:**
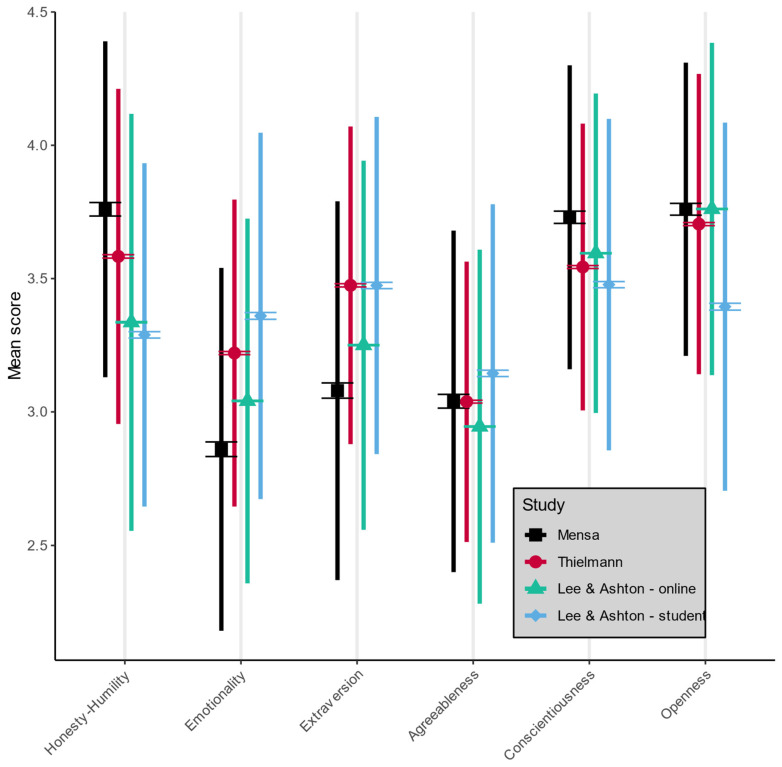
Means, standard errors, and standard deviations for HEXACO factors, displayed separately for the MENSA sample and reference samples. Means are indicated by symbols, standard errors are indicated by whiskers around the symbols, and standard deviations are indicated by colored bars.

On factor level, 15 out of 18 comparisons between gifted individuals and reference samples yielded effects of non-trivial strength (Figure 1). Table 4 provides factor level summary statistics (see Supplementary Tables S1 and S2 for more detailed statistics).

In comparison to all reference samples, Honesty-Humility was higher in gifted individuals, with effect sizes ranging from *d* = 0.28 to *d* = 0.73. Emotionality was lower in gifted individuals compared to all reference samples, with effect sizes ranging from *d* = −0.27 to *d* = −0.73. Members of the MENSA sample exhibited lower scores in Extraversion compared to all reference samples, with effect sizes ranging from *d* = −0.25 to *d* = −0.65. Comparisons between the gifted sample and reference samples yielded an inconsistent pattern for Agreeableness. MENSA members showed virtually identical values as the Thielmann sample, higher values than the online sample, and lower values than the student sample. However, effect sizes were trivial in strength, ranging from *d* = −0.16 to *d* = −0.14. Gifted persons exhibited consistently higher scores in Conscientiousness, with effect sizes ranging from *d* = 0.23 to *d* = 0.41. MENSA members showed slightly higher scores in Openness compared to the Thielmann sample and higher scores compared to the student sample, but no differences compared to the large online sample. Effect sizes ranged from *d* < 0.01 to *d* = 0.55. This pattern of results remained largely unchanged when broken down by sex (see Supplementary Figure S1 for an overview of factor scores for men and women).

On the facet level, a more nuanced picture emerged (see Table 5 and Supplementary Tables S1 and S2 for details). Gifted individuals scored higher than all reference samples in the Honesty-Humility facets of Sincerity, Fairness, and Greed-Avoidance, with effect sizes ranging from *d* = 0.18 to *d* = 0.70. The gifted sample also scored higher in Modesty compared to the online and the student sample, but not compared to the Thielmann sample.

Gifted individuals exhibited lower scores in all Emotionality facets (Fearfulness, Anxiety, Dependence, and Sentimentality yielding *d*s ranging from −0.12 to *d* = −0.73).

Across all facets, MENSA members consistently showed lower scores in Extraversion, with effect sizes ranging from *d* = −0.14 to *d* = −1.08. The only exception was the facet Social Self-Esteem, for which we found no differences between the MENSA sample and the online sample. Sociability displayed the largest effect sizes among all comparisons (*d* = −0.48 to *d* = −1.08), indicating that gifted individuals scored more than one standard deviation lower compared to the Thielmann as well as the student samples.

In Agreeableness, the pattern of results was more heterogenous. None of the facets showed effect sizes with consistent directions across samples. Effect sizes ranged from *d* = −0.31 to *d* = 0.42. Forgiveness was higher in gifted individuals compared to the Thielmann and the online sample, but lower compared to the student sample. Similarly, the facets Gentleness, Flexibility, and Patience showed small to medium effect sizes with inconsistent signs.

Gifted persons scored higher in the Conscientiousness facets of Perfectionism and Prudence compared to all reference samples (*d* = 0.25 to *d* = 0.53) but showed inconsistent and smaller effect sizes for the facets Organization and Diligence (*d* = −0.05 to *d* = 0.24).

In the Openness facets of Aesthetic Appreciation and Inquisitiveness, MENSA members exhibited higher values compared to all reference samples (*d* = 0.11 to *d* = 0.91). However, the pattern was less unambiguous for Creativity and Unconventionality (*d* = −0.17 to *d* = 0.47).

In addition, we explored differences between the German-speaking portion of the MENSA sample and the Thielmann sample that had used the German version of the HEXACO personality inventory, as well. The country-level analysis yielded results largely consistent with our main analyses. See Supplementary Table S3 for factor- and facet-level comparisons.

## 4. Discussion

Here, we provide evidence for personality factor- and facet-level differences between cognitively gifted and non-gifted samples in this first examination of the HEXACO personality model. Our findings provide several points of interest, as we describe below.

In comparison to the FFM, the HEXACO model features a sixth factor, labelled Honesty-Humility. Comparisons with all reference samples yielded meaningful effect sizes in consistent directions regarding Honesty-Humility. According to the HEXACO’s authors, “Honesty-Humility represents a tendency to treat others fairly even when one could successfully exploit them” (Lee and Ashton 2018, p. 544). Following this definition, in this study, gifted individuals exhibited a greater tendency toward prosocial and equitable behavior. This was also evident on the facet level. Gifted individuals scored higher in Sincerity, Fairness, and Greed-Avoidance. In lexical studies, adjectives that were associated with these traits include honesty, fair-mindedness, or loyalty (Ashton and Lee 2007). Modesty, on the other hand, showed smaller effect sizes and was elevated in gifted individuals only compared to the online as well as the student sample. High scores in Modesty indicate that a person is aware of their privileges and does not consider themselves to be superior to others (Lee and Ashton 2004). Since this is the first study of its kind, no previous findings are available on differences in Honesty-Humility between gifted and non-gifted individuals.

In line with our expectations, gifted individuals scored substantially lower than reference samples regarding the HEXACO factor Emotionality. This effect also emerged on the facet level. Gifted individuals scored lower in Fearfulness, Anxiety, Dependence, and Sentimentality compared to all reference samples. These results mirror research on the personality–intelligence intersection. Higher intelligence has been established as a robust predictor of lower levels of emotional maladjustment, anxiety, as well as depression (e.g., Deary et al. 2021), which has also been found to be strongly associated with the FFM factor, Neuroticism (Navrady et al. 2017). The HEXACO factor Emotionality is often likened to FFM Neuroticism which indicates emotional stability and adjustment (Ashton et al. 2014). It has been proposed that Neuroticism is negatively correlated with intelligence because negative emotionality tends to impede prefrontal brain processes which are prerequisites for complex cognition (DeYoung 2020). A recent meta-analysis also suggested lower Neuroticism in gifted individuals (*d* = −0.34, Ogurlu and Özbey 2021).

Extraversion exhibited substantially lower scores in the intellectually gifted sample compared to reference samples. The effect direction was consistent with our hypotheses. Effects were largest for comparisons with the Thielmann and the student sample. On the facet level, 11 out of 12 comparisons yielded statistically significant differences with *p*-values lower than .001. Regarding Social Self-Esteem, the comparison with the online sample showed no meaningful difference. Social Boldness, Sociability, and Liveliness consistently displayed lower scores in the gifted sample compared to reference samples. The largest effect sizes were observed for Sociability, with two out of three comparisons exhibiting differences of more than one standard deviation between mean scores. Sociability describes the disposition to seek social gatherings and celebratory events or to enjoy conversing with others (Lee and Ashton 2004). Put differently, MENSA members in this study displayed a lower inclination for social activities compared to reference samples. Previous accounts have suggested that gifted individuals show a stronger preference for intellectually stimulating, solitary activities compared to non-gifted individuals. Following this rationale, the gifted are less interested in social activities and gain greater gratification from introspection (Dijkstra et al. 2012; Likhanov et al. 2021). However, it is important to note that the available meta-analytic evidence did not indicate a meaningful linear association of Extraversion and intelligence (Stanek 2014) and no lower Extraversion scores in gifted compared to non-gifted persons (Ogurlu and Özbey 2021). These previous studies were carried out within the FFM framework, while presently the HEXACO personality model was used. Extraversion in the HEXACO model shares certain characteristics with FFM Extraversion, but it represents different facets. FFM Extraversion contains traits such as emotional toughness and bravery. Due to the different factorial solution, these traits are not part of HEXACO Extraversion but are instead captured by Emotionality (Lee and Ashton 2004). This conceptual difference may have conceivably led to results that are somewhat contrasting the existing literature.

In line with our hypotheses, Agreeableness showed small to negligible effect sizes for comparisons on the factor level. No meaningful effects were observed for comparisons with the German Thielmann sample and small effects for comparisons with the online and student samples. Effect sizes were small and inconsistent on the facet level, as well. Largest effect sizes were observed for Forgiveness, albeit they were inconsistent in direction. Gentleness, Flexibility, and Patience exhibited small to negligible effects. Thus, the intellectually gifted sample was not generally more or less agreeable than the reference samples. Agreeableness in the HEXACO framework describes the degree to which persons are willing to be patient, even-tempered, accommodating, and flexible in changing their own goals to the benefit of other persons (Lee and Ashton 2004). Previous research has come to similar conclusions: FFM Agreeableness was not found to be associated with cognitive ability on the facet level (Ackerman and Heggestad 1997; Stanek 2014). For the FFM, Politeness showed an inverse association with cognitive ability on the facet level. This FFM facet is most closely linked to the HEXACO facet Gentleness (Ludeke et al. 2019), which assesses the predisposition of people not to make harsh judgments of others and to be critical of their actions (Lee and Ashton 2004). In our study, two out of three comparisons showed that gifted individuals were indeed slightly more critical of fellow human beings compared to reference data, thus, partly conforming to these previous accounts.

Consistent with our hypotheses, MENSA members showed higher scores in Conscientiousness compared to all reference samples. On the facet level, these differences were most pronounced for Perfectionism, which describes attention to detail, and Prudence, which describes the inclination to carefully weigh and deliberate one’s course of action (Lee and Ashton 2004). Smaller and partially inconsistent effects were observed for Organization, which describes the preference for structured and orderly environments. Diligence, which speaks to a person’s eagerness to work hard to achieve one’s objectives (Lee and Ashton 2004), did not exhibit meaningful effects. The existing literature generally found no meaningful association between cognitive ability and FFM Conscientiousness scores (Stanek 2014), but results in gifted samples were somewhat inconsistent, with some studies reporting substantially higher Conscientiousness for gifted persons (e.g., Sadat et al. 2014; Biedroń 2011). Among personality dimensions, Conscientiousness is the most robust predictor of academic and professional success. In this respect, Conscientiousness shows similar predictive properties as intelligence, which is an even stronger predictor of success in these and other domains (Furnham and Cheng 2013; Ceci and Williams 1997; Ree and Earles 1992). Nevertheless, the two concepts have consistently displayed little to no overlap. A possible explanation for this seemingly paradoxical finding has become known as the compensation hypothesis. According to this hypothesis, it is argued that persons with less intelligence have to be more diligent to be equally as successful as their more intelligent peers who can “get away” with being less diligent because they work more efficiently (Chamorro-Premuzic and Furnham 2005). This may help to explain the lack of difference in the more work-ethic related aspects of Conscientiousness whereas gifted persons showed higher scores in Perfectionism and Prudence, which indicate a more detail-oriented and precise approach to tackle different tasks (Lee and Ashton 2004).

Openness showed trivial positive effects compared to the Thielmann sample and online sample but a moderate positive effect compared to the student sample, indicating that the gifted sample displayed equal to moderately elevated Openness scores when compared to reference samples. These findings only partly conformed to our expectations. On the facet level, the largest effects were observed for Inquisitiveness, which assesses the predisposition of individuals to acquire information about their surroundings. Gifted individuals exhibited higher scores in this facet compared to all reference samples. Regarding the remaining facets of Aesthetic Appreciation, Creativity, and Unconventionality, the differences were heterogeneous in direction and weaker in strength. In other words, gifted individuals were more likely to exhibit a tendency toward gaining knowledge but did not differ meaningfully from reference samples in their receptiveness for beauty, their inclination for novelty, and their disregard for societal norms. Especially regarding HEXACO Openness, the results may seem at odds with previous findings on FFM Openness. The latter has been established as the most robust correlate of intelligence among the FFM factors, displaying correlations as large as *r* = .30 (DeYoung 2020; Ackerman and Heggestad 1997); this correlation has been reported to be substantially stronger for crystallized intelligence than for fluid intelligence (Ashton et al. 2000). However, this inconsistency of our results compared to the differences that had been reported in the literature can be attributed to the differing properties of the FFM and HEXACO factor Openness. In the FFM framework, Openness is, in part, characterized by intellect and contains items that resemble self-assessed intelligence items (Stanek 2014). Some implementations of the FFM even name the factor Openness/Intellect because of its close resemblance to cognitive ability self-reports. This has also been shown empirically, where, for instance, the Big Five Aspect Scales the Openness/Intellect factor was observed to be closely linked to intellectual engagement (DeYoung et al. 2007). Within the HEXACO framework, the factor Openness differs markedly from the operationalization in the FFM. Since they were worried about a potential confounding effect of general intelligence, the HEXACO’s authors decided not to include items that are closely related to intellectual ability but chose to retain items related to intellectual curiosity (Lee and Ashton 2004). The latter is represented by the facet Inquisitiveness, in which gifted individuals displayed significantly higher scores compared to reference samples in the current study.

In summary, we provide here a first account of personality differences between gifted and non-gifted adults using the HEXACO personality model. We show substantial group differences between gifted and non-gifted individuals which suggest that personality and intelligence are not entirely independent constructs. Although, to date, the causes for these differences remain elusive, our evidence suggests that personality differences between higher and lower scorers on formal IQ tests might not behave in an identical manner across the entire cognitive ability distribution, but are conceivably differentiated according to the most extreme segments of the distribution.

### Limitations

Here, we demonstrate that intellectually gifted members of MENSA differ substantially from reference samples in pivotal aspects of their personalities. However, some limiting factors need to be discussed.

First, respondents in our survey were recruited from MENSA. This society requires applicants to score within the upmost two percentiles on a standardized test of cognitive ability in comparison to the general population. Nevertheless, even though MENSA is the world’s largest association of intellectually gifted persons, it is unclear whether it is representative of all gifted individuals. To date, little is known about personality traits that might predispose gifted individuals to consider MENSA membership. This may mean that MENSA members may not be representative for the population of gifted individuals in terms of their personality. Nevertheless, by virtue of its large membership (>145,000 members worldwide) and international network of chapters, MENSA represents an invaluable and unparalleled population of research subjects that can enable insights into psychological phenomena specific to the upper end of the intelligence distribution, that is otherwise near impossible to study.

Second, we did not have any information about the cognitive ability of participants (and, therefore, the prevalence of giftedness) in the reference datasets. In the university student sample, this percentage may be assumed to be higher than in the general population (e.g., Wai et al. 2009). If the percentage of gifted individuals in this (or any other) reference sample would indeed have been comparatively large in this sample, the differences from gifted individuals would have been underestimated. Thus, the current findings may be interpreted as conservative estimates of true effect sizes.

Although we were able to use scale-scores from the HEXACO-60 for our reference samples, internal consistencies were only available for the 100-item version of the HEXACO. This means that the reported reliabilities for the reference samples may represent slight overestimates. Prior evidence showed that reliabilities for HEXACO-60 factor scores typically ranged between α = .73 to .80 (Ashton and Lee 2009).

Finally, even though personality is generally considered to be largely stable across the human lifespan (e.g., Caspi et al. 2005), some age-related changes have been frequently noted. These include slightly declining levels of FFM Neuroticism, Extraversion, and Openness as people get older (Costa and McCrae 1997). The MENSA sample exhibited a significantly higher mean age compared to all reference samples (47.98 years vs. 32.40 years, 37.10 years, and 20.90 years). These age discrepancies could have impacted the differences between the gifted sample and the reference samples. MENSA members did exhibit lower scores in Emotionality and Extraversion, but the effect did not surface in Openness. In addition, if participant age indeed had biased the present findings, one would expect the differences to be most extreme for the biggest age gap between samples (i.e., MENSA vs. student sample). For Honesty-Humility, Emotionality, Extraversion, and Conscientiousness, the largest differences were indeed observed between these two samples, thus indicating that a certain increment of these changes could conceivably be due to age effects.

## 5. Conclusions

We provide here the first account of HEXACO-based personality differences between members of a high-intelligence society and participants that were unselected in terms of their cognitive ability from three reference samples. We show that gifted persons exhibited substantially greater Honesty-Humility, lower Emotionality, as well as higher Conscientiousness scores compared to others, whilst some differences yielding larger scores in gifted individuals were observed for Openness. Our results indicate that specific personality patterns surface at the upper end of the intelligence distribution, suggesting that intelligence and personality are not independent constructs.

## Figures and Tables

**Table 1:** Description of HEXACO factors and corresponding facets.

Factor/Facet	Description ^1^
**Honesty-Humility**	Is fair to others and does not exploit their weaknesses.
Sincerity	Is authentic and truthful in social interactions.
Fairness	Avoids defrauding others by cheating, stealing, or corruption.
Greed avoidance	Is uninterested in wealth or owning status symbols.
Modesty	Does not consider themselves to be entitled to special treatment. Is humble.
**Emotionality**	Exhibits low toughness, avoids situations that could potentially harm them.
Fearfulness	Is prone to experience fear in various situations.
Anxiety	Has the tendency to worry and to react with stress to many scenarios.
Dependence	Desires support and validation from others.
Sentimentality	Is emotionally attached to others and exhibits high empathy.
**Extraversion**	Is comfortable and energetic in social situations.
Expressiveness	Is excitable and theatrical in social interactions.
Social Boldness	Is not easily intimidated or shy.
Sociability	Likes conversations and seeks out social situations.
Liveliness	Is cheerful, high-energy, intense in interactions with others.
**Agreeableness**	Is patient and lenient with others, even if this may cause them disadvantages.
Forgiveness	Is willing to condone wrongs that others may have caused them.
Gentleness	Is uncritical toward others and tends not to evaluate them rigidly.
Flexibility	Can be easily convinced to change their plans and to cooperate.
Patience	Is composed and tolerant, not quick-tempered when things do not go as planned.
**Conscientiousness**	Takes care in their work. Approaches problems in a methodical, deliberate manner.
Organization	Prefers orderly, structured environments.
Diligence	Exerts great self-control and has a high achievement drive.
Perfectionism	Is detail-oriented in evaluating their work and spots mistakes others might overlook.
Prudence	Displays low impulsivity and weighs their options thoroughly before taking action.
**Openness**	Is curious. Prefers unconventional approaches. Expresses themselves through art.
Aesthetic Appreciation	Likes to go to museums, concerts. Enjoys beauty in many aspects of life.
Inquisitiveness	Has the urge to gather information about the world, is intellectually curious.
Creativity	Comes up with new solutions for problems, is artistically expressive.
Unconventionality	Does not conform to conventional or traditional patterns of thought.

^1^ Statements that describe a typical high scorer in the respective scales. Definitions of factors were adapted from Lee and Ashton (2018), definitions of facets were adapted from Lee and Ashton (2004).

**Table 2:** Participant age.

	*M*	*Md*	*SD*	*IQR*	*Min*	*Max*	*N*
Overall	47.98	48.00	14.91	21.00	18.00	87.00	617
Women	46.17	45.00	14.32	18.00	18.00	79.00	308
Men	49.98	50.00	15.15	24.00	18.00	87.00	309

**Table 3:** Sociodemographic sample characteristics.

	Frequency	Percentage
**Education**		
No degree	17	2.76
Post-secondary education	104	16.86
Secondary education	40	6.48
Bachelor’s degree or equivalent	153	24.80
Master’s degree or equivalent	229	37.12
Doctoral degree/PhD	69	11.18
No response	5	0.81
**MENSA intelligence assessment**		
98th percentile	165	26.74
99th percentile	238	38.57
Could not recall	214	34.68
**Country**		
Austria	39	6.32
Germany	119	19.29
Hungary	76	12.32
Switzerland	40	6.48
United Kingdom	343	55.59
**Occupation ^1^**		
Armed forces occupation	3	0.49
Clerical support worker	52	8.43
Craft and related trades worker	11	1.78
Elementary occupation	4	0.65
Manager	96	15.56
Plant and machine operator	3	0.49
Professional	302	48.95
Service and sales worker	21	3.40
Skilled agricultural, forestry or fishery worker	5	0.81
Technician or associate professional	81	13.13
No response	39	6.32

^1^ Participants’ occupation was operationalized using the International Standard Classification of Occupations (ISCO-08; International Labour Office 2012).

**Table 4:** Standardized mean differences (Cohen *d*’s) for comparisons of HEXACO factor scores.

Factor	Thielmann et al. (2020)	Lee and Ashton (2018) Online	Lee and Ashton (2018) Student
Honesty-Humility	0.28 ***	0.54 ***	0.73 ***
Emotionality	−0.62 ***	−0.27 ***	−0.73 ***
Extraversion	−0.65 ***	−0.25 ***	−0.61 ***
Agreeableness	<0.01	0.14 ***	−0.16 ***
Conscientiousness	0.35 ***	0.23 ***	0.41 ***
Openness	0.10 *	<0.01	0.55 ***

* *p* < .05; *** *p* < .001. Each line represents a comparison between the current sample of intellectually gifted MENSA members with the respective reference samples.

**Table 5:** Standardized mean differences (Cohen *d*’s) for comparisons of HEXACO facets.

Facet	Thielmann et al. (2020)	Lee and Ashton (2018) Online	Lee and Ashton (2018) Student
Honesty-Humility			
Sincerity	0.36 ***	0.56 ***	0.70 ***
Fairness	0.18 ***	0.31 ***	0.48 ***
Greed-Avoidance	0.24 ***	0.42 ***	0.59 ***
Modesty	−0.07	0.37 ***	0.22 ***
Emotionality			
Fearfulness	−0.29 ***	−0.32 ***	−0.71 ***
Anxiety	−0.12 **	−0.16 ***	−0.32 ***
Dependence	−0.58 ***	−0.14 ***	−0.58 ***
Sentimentality	−0.73 ***	−0.13 ***	−0.48 ***
Extraversion			
Social Self-Esteem	−0.29 ***	0.04	−0.29 ***
Social Boldness	−0.49 ***	−0.25 ***	−0.22 ***
Sociability	−1.03 ***	−0.49 ***	−1.08 ***
Liveliness	−0.30 ***	−0.14 ***	−0.43 ***
Agreeableness			
Forgiveness	0.42 ***	0.14 ***	−0.25 ***
Gentleness	−0.11 *	0.04	−0.31 ***
Flexibility	−0.18 ***	0.06	−0.01
Patience	−0.06	0.21 ***	0.09 *
Conscientiousness			
Organization	0.05	0.11 **	0.24 ***
Diligence	0.02	−0.05	0.07
Perfectionism	0.41 ***	0.27 ***	0.29 ***
Prudence	0.42 ***	0.25 ***	0.53 ***
Openness			
Aesthetic Appreciation	0.11 **	0.08 *	0.31 ***
Inquisitiveness	0.37 ***	0.11 ***	0.91 ***
Creativity	−0.17 ***	−0.14 ***	0.04
Unconventionality	0.05	0.00	0.47 ***

* *p* < .05; ** *p* < .01; *** *p* < .001. Each row represents three comparisons between the present sample of intellectually gifted MENSA members with the respective reference samples.

## Data Availability

The data presented in this study are available upon reasonable request from the corresponding author. The data are not publicly available for privacy reasons.

## Supplementary Materials

The following supporting information can be downloaded at: https://www.mdpi.com/article/10.3390/jintelligence10040092/s1, Table S1: Means and standard deviations for factor and facet scores; Table S2: Detailed statistics for factor and facet level comparisons; Table S3: Comparisons between Thielmann et al. (2020) and the German speaking portion of the current MENSA sample; Figure S1: Means, standard errors, and standard deviations for HEXACO factors, broken down by participant sex.
