# Supplementary material for: Is There a “Gifted Personality”? Initial Evidence for Differences between MENSA and General Population Members in the HEXACO Personality Inventory
Source: J Intell. 2022 Oct 26;10(4):92. doi: 10.3390/jintelligence10040092 (PMC9680308; doi:10.3390/jintelligence10040092)
Supplement: Supplementary file 1 [file jintelligence-10-00092-s001.zip › jintelligence-1953556-supplementary.pdf]

Supplementary Table S1. Means and standard deviations for factor and facet scores

Factor/facet	MENSA		Thielmann et al. (2020)		Lee & Ashton (2018) online		Lee & Ashton (2018) student	
	M	*SD*	*M*	*SD*	*M*	*SD*	*M*	*SD*
Honesty-Humility	3.76	0.63	3.58	0.63	3.34	0.78	3.29	0.64
Emotionality	2.86	0.68	3.22	0.58	3.04	0.68	3.36	0.69
Extraversion	3.08	0.71	3.47	0.60	3.25	0.69	3.47	0.63
Agreeableness	3.04	0.64	3.04	0.53	2.95	0.66	3.14	0.63
Conscientiousness	3.73	0.57	3.54	0.54	3.60	0.60	3.48	0.62
Openness	3.76	0.55	3.70	0.56	3.76	0.62	3.39	0.69
Honesty-Humility								
Sincerity	3.89	0.84	3.59	0.83	3.36	0.96	3.31	0.82
Fairness	3.82	1.04	3.64	1.00	3.47	1.14	3.34	1.02
Greed Avoidance	3.42	0.93	3.19	0.93	2.98	1.04	2.85	0.98
Modesty	3.82	0.9	3.88	0.81	3.46	0.99	3.63	0.89
Emotionality								
Fearfulness	2.49	0.85	2.72	0.77	2.77	0.88	3.12	0.89
Anxiety	3.4	1.11	3.52	0.95	3.57	1.04	3.73	1.00
Dependence	2.52	0.99	3.05	0.91	2.66	0.99	3.11	1.02
Sentimentality	3.1	0.9	3.64	0.73	3.22	0.89	3.52	0.87
Extraversion								
Social Self-Esteem	3.44	0.97	3.68	0.83	3.40	0.91	3.69	0.81
Social Boldness	2.95	0.93	3.33	0.78	3.17	0.90	3.15	0.91
Sociability	2.56	0.91	3.38	0.79	3.03	0.96	3.52	0.89
Liveliness	3.23	0.95	3.47	0.79	3.36	0.92	3.59	0.81
Agreeableness								
Forgivingness	2.84	1.04	2.49	0.80	2.70	1.02	3.09	1.00
Gentleness	2.95	0.82	3.03	0.70	2.91	0.84	3.19	0.78
Flexibility	2.87	0.75	2.99	0.67	2.82	0.78	2.88	0.79
Patience	3.63	0.99	3.68	0.80	3.42	1.02	3.54	1.02
Conscientiousness								
Organization	3.63	1	3.59	0.88	3.52	0.97	3.38	1.02
Diligence	3.83	0.85	3.81	0.74	3.87	0.82	3.77	0.79
Perfectionism	3.8	0.81	3.50	0.75	3.58	0.79	3.57	0.81
Prudence	3.67	0.71	3.38	0.68	3.47	0.79	3.25	0.80
Openness								

Aesthetic Appreciation	3.67	0.99	3.56	1.01	3.58	1.01	3.34	1.09
Inquisitiveness	3.98	0.79	3.67	0.83	3.88	0.89	3.06	1.06
Creativity	3.61	0.88	3.74	0.77	3.74	0.90	3.57	0.99
Unconventionality	3.82	0.69	3.79	0.65	3.82	0.71	3.48	0.73
n	**615**		**9,491**		**100,318**		**2,868**	

Supplementary Table S2. Detailed statistics for factor and facet level comparisons

Study	Factor/facet	*t*	*df*[1]	Mean difference [95% *CI*]	*p*	*d*
Thielmann et al. (2020)	Honesty-Humility	-6.75	695.55	-0.18 [-0.23; -0.13]	< .000001	0.28
Thielmann et al. (2020)	Emotionality	12.86	672.25	0.36 [0.31; 0.42]	< .000001	-0.62
Thielmann et al. (2020)	Extraversion	13.48	671.23	0.39 [0.34; 0.45]	< .000001	-0.65
Thielmann et al. (2020)	Agreeableness	-0.06	668.72	0.00 [-0.05; 0.05]	.950181	0.00
Thielmann et al. (2020)	Conscientiousness	-7.89	686.76	-0.19 [-0.23; -0.14]	< .000001	0.35
Thielmann et al. (2020)	Openness	-2.42	700.04	-0.06 [-0.10; -0.01]	.015744	0.10
			**Honesty-Humility**			
Thielmann et al. (2020)	Sincerity	-8.69	694.68	-0.30 [-0.37; -0.24]	< .000001	0.36
Thielmann et al. (2020)	Fairness	-4.09	689.52	-0.18 [-0.26; -0.09]	.000049	0.18
Thielmann et al. (2020)	Greed Avoidance	-5.88	696.55	-0.23 [-0.30; -0.15]	< .000001	0.24
Thielmann et al. (2020)	Modesty	1.58	679.33	0.06 [-0.01; 0.13]	.114440	-0.07
			**Emotionality**			
Thielmann et al. (2020)	Fearfulness	6.44	680.11	0.23 [0.16; 0.30]	< .000001	-0.29
Thielmann et al. (2020)	Anxiety	2.53	673.43	0.12 [0.03; 0.21]	.011772	-0.12
Thielmann et al. (2020)	Dependence	12.94	682.91	0.53 [0.45; 0.61]	< .000001	-0.58
Thielmann et al. (2020)	Sentimentality	14.62	666.84	0.54 [0.47; 0.61]	< .000001	-0.73

**Extraversion**						
Thielmann et al. (2020)	Social Self-Esteem	6.02	673.03	0.24 [0.16; 0.32]	< .000001	-0.29
Thielmann et al. (2020)	Social Boldness	10.03	671.00	0.38 [0.31; 0.46]	< .000001	-0.49
Thielmann et al. (2020)	Sociability	21.84	674.79	0.82 [0.75; 0.89]	< .000001	-1.03
Thielmann et al. (2020)	Liveliness	6.13	670.36	0.24 [0.16; 0.32]	< .000001	-0.30
**Agreeableness**						
Thielmann et al. (2020)	Forgivingness	-8.12	661.90	-0.35 [-0.43; -0.26]	< .000001	0.42
Thielmann et al. (2020)	Gentleness	2.24	673.53	0.08 [0.01; 0.14]	.025667	-0.11
Thielmann et al. (2020)	Flexibility	3.85	679.07	0.12 [0.06; 0.18]	.000131	-0.18
Thielmann et al. (2020)	Patience	1.13	666.95	0.05 [-0.03; 0.13]	.257179	-0.06
**Conscientiousness**						
Thielmann et al. (2020)	Organization	-1.03	677.43	-0.04 [-0.12; 0.04]	.304205	0.05
Thielmann et al. (2020)	Diligence	-0.47	675.67	-0.02 [-0.09; 0.05]	.640808	0.02
Thielmann et al. (2020)	Perfectionism	-9.09	683.16	-0.30 [-0.37; -0.24]	< .000001	0.41
Thielmann et al. (2020)	Prudence	-9.78	688.10	-0.29 [-0.35; -0.23]	< .000001	0.42
**Openness**						
Thielmann et al. (2020)	Aesthetic Appreciation	-2.63	699.96	-0.11 [-0.19; -0.03]	.008711	0.11

Thielmann et al. (2020)	Inquisitiveness	-9.41	705.31	-0.31 [-0.37; -0.25]	< .000001	0.37
Thielmann et al. (2020)	Creativity	3.61	676.91	0.13 [0.06; 0.20]	.000335	-0.17
Thielmann et al. (2020)	Unconventionality	-1.17	686.12	-0.03 [-0.09; 0.02]	.241510	0.05
Lee & Ashton (2018) online	Honesty-Humility	-16.60	625.64	-0.42 [-0.47; -0.37]	< .000001	0.54
Lee & Ashton (2018) online	Emotionality	6.59	621.63	0.18 [0.13; 0.24]	< .000001	-0.27
Lee & Ashton (2018) online	Extraversion	5.94	621.17	0.17 [0.11; 0.23]	< .000001	-0.25
Lee & Ashton (2018) online	Agreeableness	-3.67	622.12	-0.09 [-0.15; -0.04]	.000266	0.14
Lee & Ashton (2018) online	Conscientiousness	-5.85	622.34	-0.13 [-0.18; -0.09]	< .000001	0.23
Lee & Ashton (2018) online	Openness	0.05	623.70	0.00 [-0.04; 0.04]	.962014	0.00
		**Honesty-Humility**				
Lee & Ashton (2018) online	Sincerity	-15.68	623.78	-0.53 [-0.60; -0.47]	< .000001	0.56
Lee & Ashton (2018) online	Fairness	-8.36	623.11	-0.35 [-0.43; -0.27]	< .000001	0.31
Lee & Ashton (2018) online	Greed Avoidance	-11.57	623.42	-0.44 [-0.51; -0.36]	< .000001	0.42
Lee & Ashton (2018) online	Modesty	-9.89	623.05	-0.36 [-0.43; -0.29]	< .000001	0.37
		**Emotionality**				
Lee & Ashton (2018) online	Fearfulness	8.14	622.15	0.28 [0.21; 0.35]	< .000001	-0.32
Lee & Ashton (2018) online	Anxiety	3.69	620.63	0.17 [0.08; 0.25]	.000239	-0.16

Lee & Ashton (2018) online	Dependence	3.38	621.56	0.14 [0.06; 0.21]	.000776	-0.14
Lee & Ashton (2018) online	Sentimentality	3.31	621.45	0.12 [0.05; 0.19]	.000998	-0.13
**Extraversion**						
Lee & Ashton (2018) online	Social Self-Esteem	-1.00	620.71	-0.04 [-0.12; 0.04]	.318145	0.04
Lee & Ashton (2018) online	Social Boldness	5.94	621.09	0.22 [0.15; 0.30]	< .000001	-0.25
Lee & Ashton (2018) online	Sociability	12.76	622.34	0.47 [0.40; 0.54]	< .000001	-0.49
Lee & Ashton (2018) online	Liveliness	3.41	621.10	0.13 [0.06; 0.21]	.000683	-0.14
**Agreeableness**						
Lee & Ashton (2018) online	Forgivingness	-3.30	621.30	-0.14 [-0.22; -0.06]	.001036	0.14
Lee & Ashton (2018) online	Gentleness	-1.11	622.00	-0.04 [-0.10; 0.03]	.266152	0.04
Lee & Ashton (2018) online	Flexibility	-1.48	622.23	-0.05 [-0.10; 0.01]	.138258	0.06
Lee & Ashton (2018) online	Patience	-5.32	621.95	-0.21 [-0.29; -0.13]	< .000001	0.21
**Conscientiousness**						
Lee & Ashton (2018) online	Organization	-2.64	621.14	-0.11 [-0.19; -0.03]	.008447	0.11
Lee & Ashton (2018) online	Diligence	1.10	621.09	0.04 [-0.03; 0.11]	.270885	-0.05
Lee & Ashton (2018) online	Perfectionism	-6.59	621.16	-0.22 [-0.28; -0.15]	< .000001	0.27
Lee & Ashton (2018) online	Prudence	-6.88	623.29	-0.20 [-0.25; -0.14]	< .000001	0.25

**Openness**						
Lee & Ashton (2018) online	Aesthetic Appreciation	-2.13	621.79	-0.09 [-0.16; -0.01]	.033906	0.08
Lee & Ashton (2018) online	Inquisitiveness	-3.10	623.64	-0.10 [-0.16; -0.04]	.002006	0.11
Lee & Ashton (2018) online	Creativity	3.64	621.97	0.13 [0.06; 0.20]	.000296	-0.14
Lee & Ashton (2018) online	Unconventionality	0.01	622.06	0.00 [-0.05; 0.06]	.994141	0.00
Lee & Ashton (2018) student	Honesty-Humility	-16.76	910.14	-0.47 [-0.53; -0.42]	< .000001	0.73
Lee & Ashton (2018) student	Emotionality	16.52	903.02	0.50 [0.44; 0.56]	< .000001	-0.73
Lee & Ashton (2018) student	Extraversion	12.73	835.52	0.39 [0.33; 0.45]	< .000001	-0.61
Lee & Ashton (2018) student	Agreeableness	3.68	891.50	0.10 [0.05; 0.16]	.000245	-0.16
Lee & Ashton (2018) student	Conscientiousness	-9.81	953.91	-0.25 [-0.30; -0.20]	< .000001	0.41
Lee & Ashton (2018) student	Openness	-14.24	1072.86	-0.37 [-0.42; -0.31]	< .000001	0.55
**Honesty-Humility**						
Lee & Ashton (2018) student	Sincerity	-15.50	880.38	-0.58 [-0.65; -0.50]	< .000001	0.70
Lee & Ashton (2018) student	Fairness	-10.53	882.96	-0.48 [-0.58; -0.39]	< .000001	0.48
Lee & Ashton (2018) student	Greed Avoidance	-13.76	933.07	-0.57 [-0.66; -0.49]	< .000001	0.59
Lee & Ashton (2018) student	Modesty	-4.87	888.60	-0.19 [-0.27; -0.12]	.000001	0.22
**Emotionality**						

Lee & Ashton (2018) student	Fearfulness	16.45	927.78	0.63 [0.55; 0.70]	< .000001	-0.71
Lee & Ashton (2018) student	Anxiety	6.72	841.87	0.33 [0.23; 0.42]	< .000001	-0.32
Lee & Ashton (2018) student	Dependence	13.41	914.66	0.59 [0.51; 0.68]	< .000001	-0.58
Lee & Ashton (2018) student	Sentimentality	10.67	876.68	0.42 [0.35; 0.50]	< .000001	-0.48
			**Extraversion**			
Lee & Ashton (2018) student	Social Self-Esteem	5.86	805.91	0.25 [0.16; 0.33]	< .000001	-0.29
Lee & Ashton (2018) student	Social Boldness	4.94	885.19	0.20 [0.12; 0.28]	.000001	-0.22
Lee & Ashton (2018) student	Sociability	23.94	884.76	0.96 [0.89; 1.04]	< .000001	-1.08
Lee & Ashton (2018) student	Liveliness	8.68	816.33	0.36 [0.28; 0.44]	< .000001	-0.43
			**Agreeableness**			
Lee & Ashton (2018) student	Forgivingness	5.38	872.13	0.25 [0.16; 0.34]	< .000001	-0.25
Lee & Ashton (2018) student	Gentleness	6.64	865.79	0.24 [0.17; 0.31]	< .000001	-0.31
Lee & Ashton (2018) student	Flexibility	0.18	928.36	0.01 [-0.06; 0.07]	.853324	-0.01
Lee & Ashton (2018) student	Patience	-2.11	915.99	-0.09 [-0.18; -0.01]	.035394	0.09
			**Conscientiousness**			
Lee & Ashton (2018) student	Organization	-5.57	906.32	-0.25 [-0.34; -0.16]	< .000001	0.24
Lee & Ashton (2018) student	Diligence	-1.52	856.80	-0.06 [-0.13; 0.02]	.128310	0.07
Lee & Ashton (2018) student	Perfectionism	-6.46	895.29	-0.23 [-0.30; -0.16]	< .000001	0.29

Lee & Ashton (2018) student	Prudence	-12.91	973.69	-0.42 [-0.48; -0.35]	< .000001	0.53
			**Openness**			
Lee & Ashton (2018) student	Aesthetic Appreciation	-7.39	963.07	-0.33 [-0.42; -0.24]	< .000001	0.31
Lee & Ashton (2018) student	Inquisitiveness	-24.59	1138.31	-0.92 [-0.99; -0.85]	< .000001	0.91
Lee & Ashton (2018) student	Creativity	-1.00	977.90	-0.04 [-0.12; 0.04]	.319524	0.04
Lee & Ashton (2018) student	Unconventionality	-10.94	936.47	-0.34 [-0.40; -0.28]	< .000001	0.47

Each row represents three comparisons between the current sample of intellectually gifted MENSA members with the respective reference samples. [1]) Degrees of freedom calculated using the Welch–Satterthwaite equation for pooled degrees of freedom.

Supplementary Table S3. Comparisons between Thielmann et al. (2020) and the German speaking portion of the current MENSA sample

Study	Factor/facet	*t*	*df*[1]	Mean difference [95% *CI*]	*p*	*d*
Thielmann et al. (2020)	Honesty-Humility	-3.19	203.92	-0.15 [-0.24; -0.06]	.001657	0.23
Thielmann et al. (2020)	Emotionality	7.82	202.24	0.37 [0.28; 0.46]	< .000001	-0.64
Thielmann et al. (2020)	Extraversion	8.06	201.94	0.40 [0.31; 0.50]	< .000001	-0.68
Thielmann et al. (2020)	Agreeableness	-0.98	202.50	-0.04 [-0.13; 0.04]	.326919	0.08
Thielmann et al. (2020)	Conscientiousness	-2.11	203.31	-0.09 [-0.17; -0.01]	.035774	0.16
Thielmann et al. (2020)	Openness	-1.45	205.29	-0.06 [-0.13; 0.02]	.147938	0.10
			**Honesty-Humility**			
Thielmann et al. (2020)	Sincerity	-3.65	203.20	-0.23 [-0.36; -0.11]	.000330	0.28
Thielmann et al. (2020)	Fairness	-1.13	203.30	-0.09 [-0.24; 0.06]	.258050	0.09
Thielmann et al. (2020)	Greed Avoidance	-4.12	203.60	-0.29 [-0.43; -0.15]	.000054	0.31
Thielmann et al. (2020)	Modesty	0.46	202.88	0.03 [-0.10; 0.15]	.648936	-0.04
			**Emotionality**			
Thielmann et al. (2020)	Fearfulness	5.92	203.71	0.34 [0.22; 0.45]	< .000001	-0.44
Thielmann et al. (2020)	Anxiety	1.18	201.78	0.10 [-0.06; 0.26]	.239362	-0.10
Thielmann et al. (2020)	Dependence	8.50	203.53	0.58 [0.45; 0.72]	< .000001	-0.64

Thielmann et al. (2020)	Sentimentality	7.15	201.10	0.47 [0.34; 0.60]	< .000001	-0.65
**Extraversion**						
Thielmann et al. (2020)	Social Self-Esteem	2.50	201.49	0.18 [0.04; 0.32]	.013221	-0.22
Thielmann et al. (2020)	Social Boldness	6.08	202.43	0.38 [0.26; 0.51]	< .000001	-0.49
Thielmann et al. (2020)	Sociability	12.43	201.99	0.82 [0.69; 0.95]	< .000001	-1.04
Thielmann et al. (2020)	Liveliness	5.41	202.34	0.35 [0.22; 0.48]	< .000001	-0.44
**Agreeableness**						
Thielmann et al. (2020)	Forgivingness	-3.13	202.20	-0.21 [-0.34; -0.08]	.001993	0.26
Thielmann et al. (2020)	Gentleness	-0.26	202.81	-0.01 [-0.12; 0.09]	.795955	0.02
Thielmann et al. (2020)	Flexibility	1.97	203.53	0.10 [0.00; 0.20]	.049947	-0.15
Thielmann et al. (2020)	Patience	-2.15	202.06	-0.14 [-0.28; -0.01]	.032465	0.18
**Conscientiousness**						
Thielmann et al. (2020)	Organization	0.63	201.79	0.05 [-0.10; 0.20]	.529150	-0.05
Thielmann et al. (2020)	Diligence	2.21	202.36	0.13 [0.01; 0.25]	.027890	-0.18
Thielmann et al. (2020)	Perfectionism	-5.34	202.79	-0.31 [-0.43; -0.20]	< .000001	0.42
Thielmann et al. (2020)	Prudence	-1.92	203.44	-0.10 [-0.20; 0.00]	.056367	0.14
**Openness**						

Thielmann et al. (2020)	Aesthetic Appreciation	-0.53	204.44	-0.04 [-0.18; 0.10]	.593235	0.04
Thielmann et al. (2020)	Inquisitiveness	-6.35	204.69	-0.37 [-0.49; -0.26]	< .000001	0.44
Thielmann et al. (2020)	Creativity	1.91	203.49	0.11 [0.00; 0.23]	.057933	-0.14
Thielmann et al. (2020)	Unconventionality	-0.28	203.69	-0.01 [-0.11; 0.08]	.779119	0.02

Each row represents three comparisons between the current sample of intellectually gifted MENSA members with the respective reference samples. [1]) Degrees of freedom calculated using the Welch–Satterthwaite equation for pooled degrees of freedom.

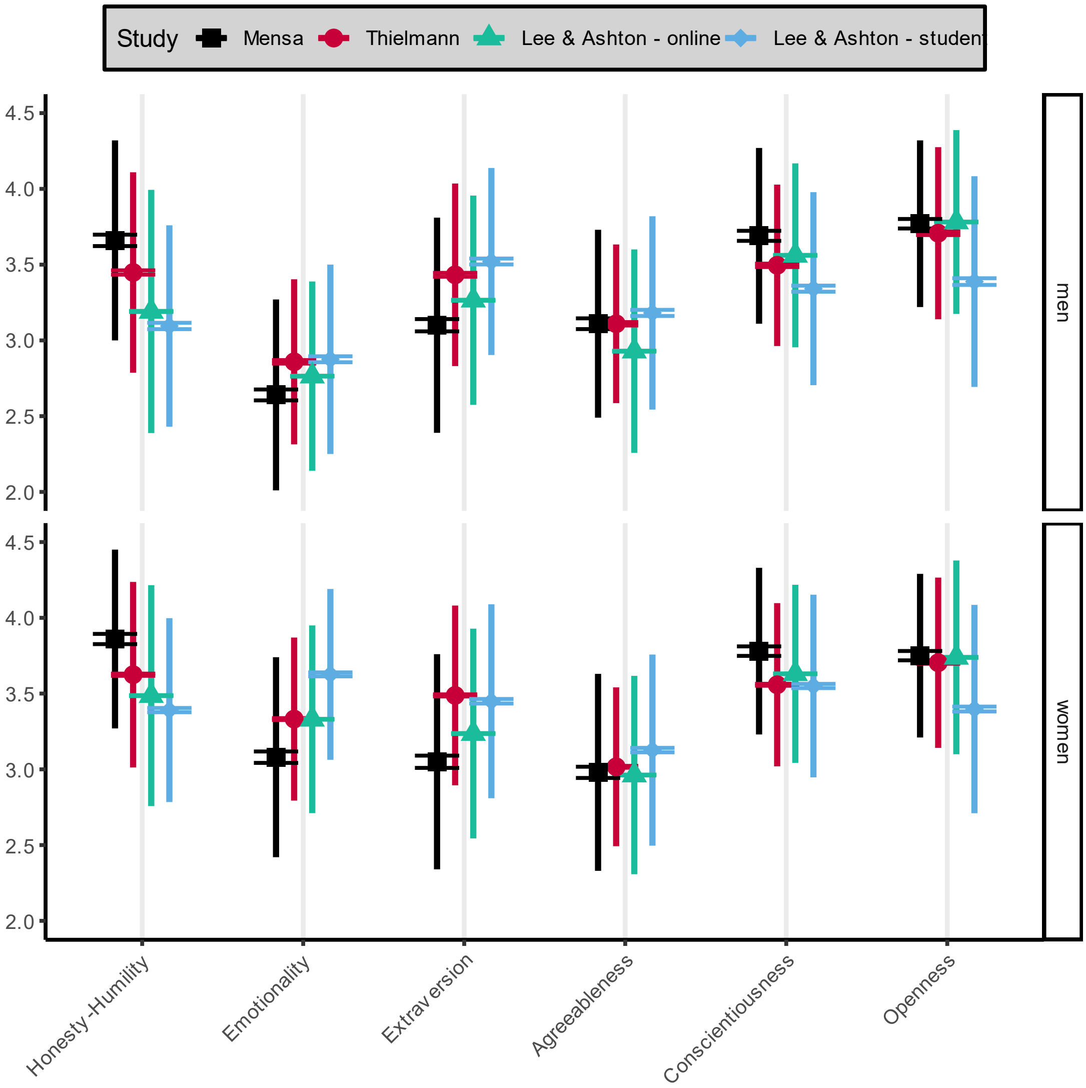

Supplementary Figure S1. Means, standard errors, and standard deviations for HEXACO factors, broken down by participant sex. Means are indicated by symbols, standard errors are indicated by whiskers around the symbols, and standard deviations are indicated by colored bars.
